# Supplementary material for: The interactome of intact mitochondria by cross-linking mass spectrometry provides evidence for coexisting respiratory supercomplexes
Source: Mol Cell Proteomics. 2017 Dec 8;17(2):216–32. doi: 10.1074/mcp.RA117.000470 (PMC5795388; doi:10.1074/mcp.RA117.000470)
Supplement: Supplemental Data [file supp_RA117.000470_133922_0_supp_23978_fzffwf.zip › spectra_annotation/mito_DR_spectra_annotation/16-1-2-1-20-1.pdf]

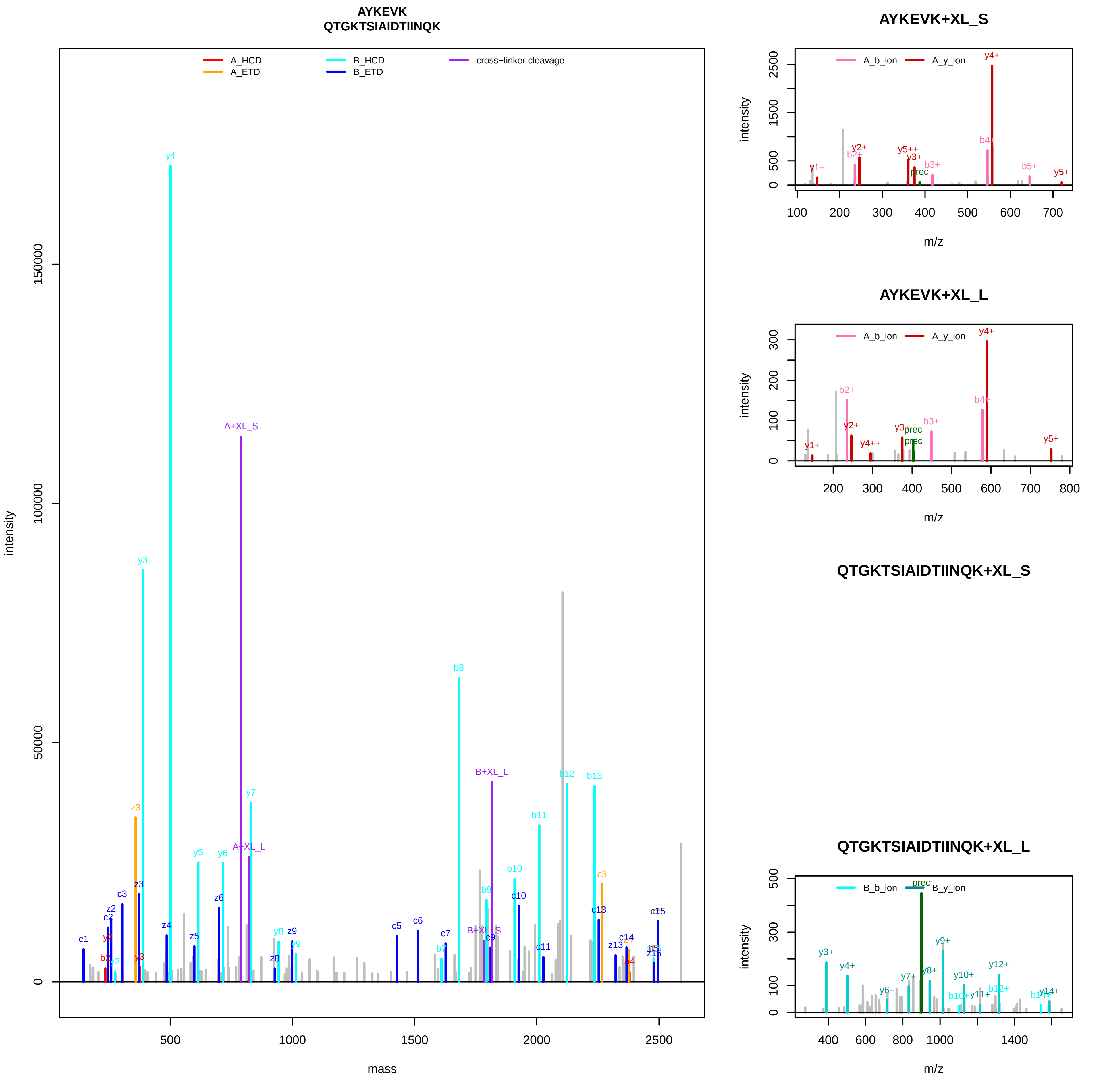

AYKEVK
QTGKTSIAIDTIINQK
A_HCD
A_ETD
B_HCD
B_ETD
cross−linker cleavage
intensity
mass
0
50000
100000
150000
500
1000
1500
2000
2500
y4
A+XL_S
y3
b8
B+XL_L
b12
b13
y7
z3
A+XL_L
y5
y6
b10
b11
c3
AYKEVK+XL_S
A_b_ion
A_y_ion
intensity
m/z
0
500
1500
2500
100
200
300
400
500
600
700
AYKEVK+XL_L
A_b_ion
A_y_ion
intensity
m/z
0
100
200
300
200
300
400
500
600
700
800
QTGKTSIAIDTIINQK+XL_S
QTGKTSIAIDTIINQK+XL_L
B_b_ion
B_y_ion
intensity
m/z
0
100
300
500
400
600
800
1000
1400
